# Association between the prudent dietary pattern and blood pressure in Chinese adults is partially mediated by body composition

**DOI:** 10.3389/fnut.2023.1131126

**Published:** 2023-03-23

**Authors:** Mengxue Chen, Yujie Xu, Xiaoyu Wang, Shufang Shan, Guo Cheng

**Affiliations:** ^1^West China School of Public Health and West China Fourth Hospital, Sichuan University, Chengdu, China; ^2^Laboratory of Molecular Translational Medicine, Center for Translational Medicine, Key Laboratory of Birth Defects and Related Diseases of Women and Children (Sichuan University), Ministry of Education, Department of Pediatrics, West China Second University Hospital, Sichuan University, Chengdu, Sichuan, China

**Keywords:** blood pressure, dietary pattern, factor analysis, body mass index, mediation effect

## Abstract

High blood pressure or hypertension is one of the major risks of cardiovascular disease, which is the leading cause of death in China. This study aimed to assess the relationship between dietary patterns and blood pressure among Chinese adults. Using factor analysis of 66-item food frequency questionnaire to identify dietary patterns. Systolic blood pressure (SBP) and diastolic blood pressure (DBP) were measured according to standardized guidelines. Multivariate linear regressions were performed in 6849 Chinese adults (46.5% female) aged 21–70 years considering sociodemographic characteristics, lifestyle behaviors, and anthropometry data. The vegetable-rich pattern, animal-food pattern, and prudent dietary pattern were identified. After adjustment for potential confounders including age, gender, alcohol consumption, smoking status, energy intake, and physical activity, only prudent dietary pattern was negatively related to SBP (*β* = −2.30, *p* for trend =0.0003) and DBP (*β* = −1.44, *p* for trend =0.0006). Body mass index, waist circumstance and body fat percentage explained, respectively, 42.5%/47.8, 14.8%/17.6 and 26.0%/29.1% of the association between prudent pattern and SBP/DBP in mediation analysis. There were no association were observed between other dietary patterns and blood pressure. In conclusion, Prudent dietary pattern was associated with lower SBP and DBP among Southwest Chinese and this association was partially explained by body composition.

## Introduction

Hypertension is the primary risk factor for cardiovascular disease (CVD) (1), which is the leading cause of death in China (2). Recently, the prevalence of hypertension in China increased from 24.9% in 2004 to 38.1% in 2018 (3), and the prevalence was as high as 44.7% among adults aged 35–75 from 2014–2017 (4). In addition, the awareness, treatment, and control rates of hypertension are at a relatively low level (3, 4), increasing the burden of CVD morbidity and mortality. Thus, an improved understanding of hypertension prevention and control from the perspective of modifiable risk factors is of significant public health interest (5). Accumulating studies have suggested that diet is an established risk factor influencing blood pressure (6, 7). Besides these associations of individual nutrients or foods and food groups with blood pressure (8–11), clinical trials found that total dietary patterns, which describes the combinations of foods and nutrients consumed in totality by individuals, were better on lowing blood pressure (12). Recent observation studies also found that dietary patterns were associated with the prevalence of hypertension (13–18), for instance, traditional or western dietary pattern was associated with high blood pressure. However, these previous studies among Chinese adults have been based on earlier data (from 2002 to 2013) (16–18) or have limited study sample (ranging from 2,518 to 3,591) (16, 17), discouraging the optimal practical value for such evidence. An updated investigation on dietary pattern and blood pressure based on a larger sample of Chinese adults is urgently needed.

Notably, body composition, indicated body mass index (BMI), waist circumstance (WC), and percentage of body fat (BF%) could be attributed to dietary patterns and simultaneously has been indicated to be positively associated with blood pressure (19–21). In that way, body composition might confound or mediate the impact of dietary pattens on hypertension. For example, Shi et al. found the association between dietary pattern and hypertension was attenuated after the addition of BMI to the model (22). Livingstone et al. reported the relevance of dietary pattern with hypertension was stronger in individuals with overweight/obese (23). A recent study considered the effect of the Dietary Approaches to Stop Hypertension (DASH) diet for hypertension may be fully influenced by BMI (24). These studies suggest the relationship between diet pattern, body composition, and hypertension is complex and further research is needed. Over the past decades, China has been under a drastic transition in the diet, leading to unfavorable changes in dietary pattern characterized with increasing consumption of animal-source food, oils, and sugar-sweetened beverages (25). Given the emerging epidemic of hypertension as well as obesity, it is valuable to consider the potential effect of body composition in the dietary pattern-blood pressure association.

Therefore, using the cross-sectional data 2013 to 2018 from a population-based cohort among Southwest Chinese adults, we aimed to determine the association between dietary patterns derived by factor analysis and blood pressure and investigate the possible mediation effect of body composition.

## Methods

### Study sample

We obtained data from the Nutrition and Health in Southwest China (NHSC) 2013–2018 baseline survey to investigate the role of diet, lifestyle, genetic background, and their interactions on non-communicable diseases among Chinese adults. Details on study protocol have been described previously (26). In brief, 54 study sites (23 communities and 31 villages) from Sichuan, Guizhou, Yunnan provinces were included until 2018. At each site, we used a 2-stage (household person) sampling. Individuals who accepted the invitation to participate were invited to the study center for interviews. All assessments of participants in the NHSC Study have included questionnaires, anthropometric measurements, medical examinations, biochemical measurements, and face-to-face interviews by trained investigators about nutrition-related behaviors, lifestyles, and social status. To facilitate follow-up, participants who volunteered to participate, and signed an informed consent form were included in the final study. The study was approved by the Research Ethics Committee of Sichuan University. All participants had signed informed written consent.

**Figure 1:**
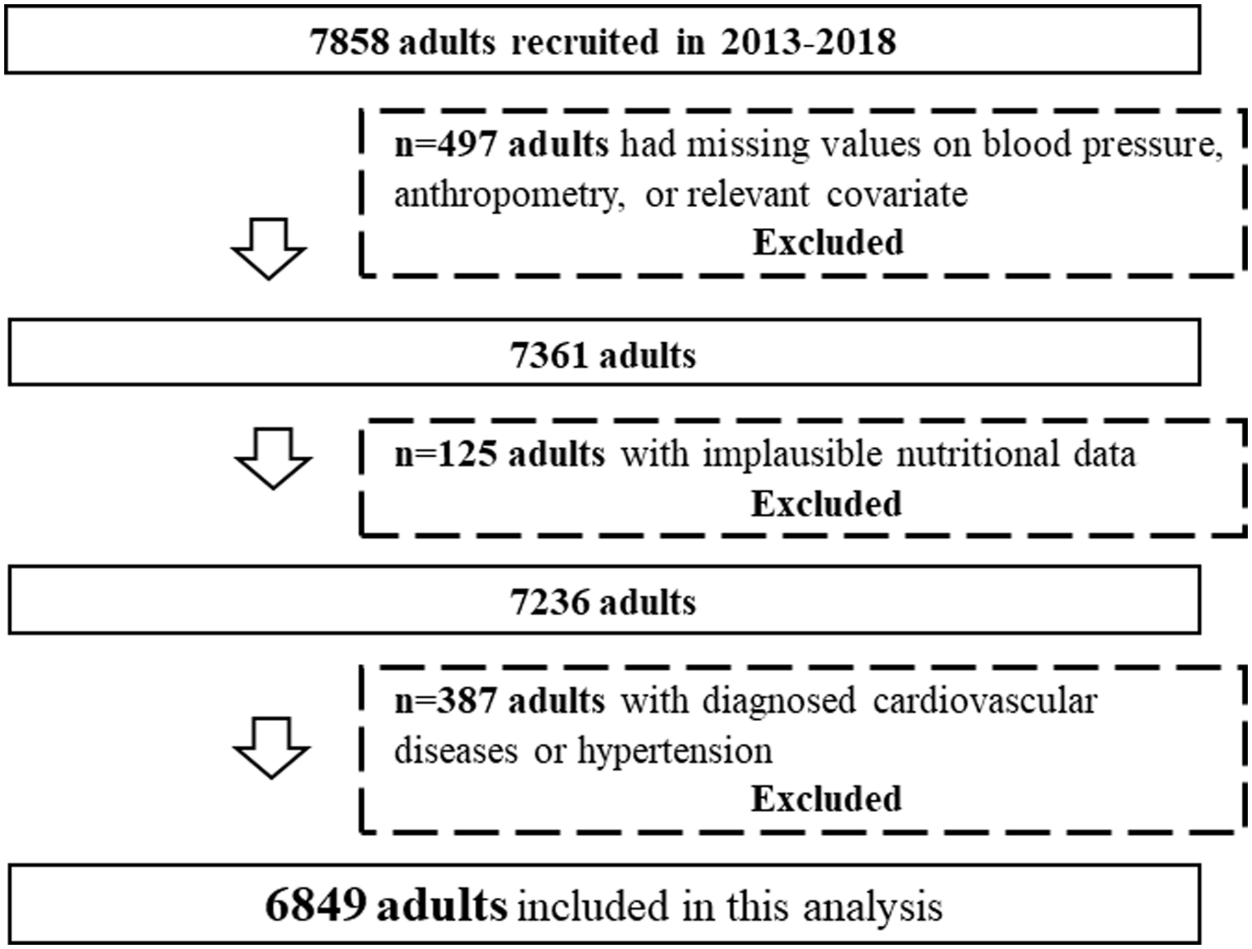
Flowchart for the study sample.

This cross-sectional study used the data of 7,858 adults. Among them, adults with incomplete information regarding blood pressure data or other confounding variables (*n* = 497), with implausible energy intake (i.e., >4,200 or < 800 kcal/d for men and > 3,500 or < 500 kcal/d for women) (27) (*n* = 125), and with diagnosed cardiovascular diseases or hypertension at baseline (*n* = 387) were excluded. Finally, 6,849 participants (3,664 men and 3,185 women) aged 21–70 y were included in the present analysis (Figure 1).

### Dietary intake data

Usual dietary intake information during the previous year was obtained from 66-item food frequency questionnaire (FFQ), this FFQ was developed based on the validated questionnaire (28, 29) that was used in the 2002 China National Nutrition and Health Survey, and be additionally modified added a few individual food groups, e.g., coffee (30) and tea (31), as their suspected biological effects; split some food groups into more clearly precise categories, e.g., wheat flour products were split into noodles, steamed rolls, dumplings and steamed stuffed bun. Participants were asked to recall the frequency of the consumption of each food item using daily, weekly, monthly, annually, or never and the estimated portion size, using local weight in China, i.e., 1 liang = 50 g or natural units, e.g., bowls. To enhance the accuracy of the estimated serving sizes, standard tableware including bowls, plates, and glasses were provided. Total energy intake and nutrients intake were assessed based on the China Food Composition Table (32).

### Dietary pattern derivation

The food items from FFQ were categorized into main food groups based on similar sources, nutrient profiles (32), or hypothesized biological effects. As a result, 24 food groups were entered into the analysis in absolute weights (Table 1). Factor analysis was conducted in the SAS software (version 9.4, SAS Institute Inc., Cary, NC, United States.) with the PROC FACTOR procedure to identify dietary patterns. Kaiser-Meyer-Olkin test was 0.67 and the Bartlett’s test of sphericity reaching statistical significance (*p* < 0.0001) indicated suitability of food intake data for factor analysis. The factors were orthogonally rotated to achieve a simpler structure with greater interpretability. Three factors (dietary patterns) to be retained were determined based on eigenvalues >1, the inspection of scree plot, and the interpretability of factors. Factor loadings reflect the correlations of each food group with the corresponding dietary pattern. Food groups with factor loadings ≥0.30 or ≤ −0.30 were considered as the most important contributors to each factor (33), and thus descriptive of that dietary pattern. Furthermore, the factor score for each pattern was constructed by summing standardized intakes of each food group weighted by their factor loadings, so that higher factor scores indicated better adherence to dietary patterns.

**Table 1:** Food or food groups used in the dietary pattern analysis.

Food or food groups	Food items
Rice and its products	Rice, brown rice, black rice, sticky rice, rice noodles
Noodles	Noodles, pasta
Wheat and its products	steamed bun, steamed stuffed bun
Whole grains products	Graham Bread, multigrain biscuits
Ethnic foods	Tangyuan, spring rolls, mooncake, mung bean cake
Corn	Corn
Tubers	Potato, cassava, taro, yam
Sweet potato	Sweet potato
Legumes and its products	Dried legumes, tofu, soya-bean milk, dried bean curd, mung bean, red bean
Dark Vegetables	Leafy and flowering vegetable, aquatic vegetable
Light-colored vegetables	Root vegetable, leguminous vegetable and sprout, cucurbitaceous and solanaceous vegetable
Fungi and algae	Mushroom, agaric, tremella, laver, sea-tangle
Fruits	Kernel fruit, drupe fruit, berry, orange fruit, tropic fruit, melons
Nuts	Walnut, melon seeds, cashew, hazelnut, almond, pistachio
Meat and its products	Pork, beef, mutton, rabbit meat, processed pork, sausage
Poultry and its products	Chicken, duck, goose, turkey, pigeon
Animal organ	Animal heart, animal kidney, animal liver, animal lung
Fish and shellfish	Fish, shrimp, crab, shellfish
Eggs	Chicken eggs, duck eggs, goose eggs, partridge eggs
Preserved eggs	Salt eggs, year eggs
Pickled vegetables	Fermented soybean curd, salted vegetables, Chinese sauerkraut
Dairy products	Milk, dried milk, yoghurt, cheese
Tea and coffee	Green tea, black tea, oolong, coffee
Beverages	Carbonated drink, fruit juice, Yakult

### Blood pressure measurement

Participants’ systolic blood pressure (SBP) and diastolic blood pressure (DBP) were measured twice by trained nurses with a standard mercury sphygmomanometer in their right arm, after a rest for about 5–10 min in the sitting position. SBP and DBP were recorded for each participant. If the difference in SBP and/or DBP between the first and second measurement was larger than 5 mmHg, the third measurement was taken. The average SBP and DBP were calculated from the nearest two values.

### Covariates

Detailed information on participants’ socio-demographic characteristics such as sex, age (years), and lifestyle behaviors including smoking status (current smokers, former smokers who quit smoking or non-current smokers who never smoked), alcohol consumption (yes: at least once in the past year; or no) was collected from a questionnaire-based interview. The physical activity was estimated in metabolic equivalents-hours per week (MET-hours/week) from moderate-to-vigorous physical activity (MVPA) (34).

Anthropometric measurements were performed by trained investigators according to standard procedures, with the subjects dressed lightly and barefoot. Height and weight were measured to the nearest 0.1 cm and 0.1 kg, respectively, using an Ultrasonic instrument (Weight and Height Instrument DHM-30; Dingheng Ltd., Zhengzhou Province, China). WC was measured at a point midway between the lowest rib margin and the iliac crest in a horizontal plane using non-elastic tape to the nearest 0.1 cm. All anthropometric measurements were performed twice for each participant. BMI was calculated as weight in kilograms divided by height in meters squared of the individual. %BF was calculated from BMI and WC using the equations from Liu, X. et al. (35) Body composition data were used as continuous variable in this analysis.

### Statistical analysis

All statistical analyses were conducted using SAS procedures (version 9.4, SAS Institute Inc., Cary, NC, United States.) and R version 4.1.1. Results were considered statistically significant when a two-sided *value of p* <0.05.

Dietary pattern scores were grouped into tertiles (T1, T2, and T3) to obtain three categories indicating a low, moderate, and high adherence to a dietary pattern. Socio-demographic characteristics were described across tertiles in each dietary pattern. Difference in continuous variables was estimated using the generalized linear model, and categorical variables were compared by the chi-square test. To further investigate the nutrient composition of each dietary pattern, linear correlations were performed with confounders in regards to the association between nutrients intakes and dietary patterns including age, gender, and energy intake.

We used multivariate linear regression models (PROC GLM procedure in SAS software) to explore the relationship of dietary pattern scores (continuous variable) with SBP and DBP. Three models were used in our study: model 1 was adjusted for age and gender, Model 2 was further adjusted for smoking status, alcohol consumption, energy intake and physical activity, and models 3–5 was additionally adjusted for body composition (In order, BMI, WC, BF%) to determine if the effects were independent of body size.

Furthermore, we created linear models to measure the association between dietary pattern scores (exposure), blood pressure (outcome), and body composition (mediator). Mediation analysis was performed only if the mediator variable was significantly associated with both the exposure and outcome. Mediation by body composition was estimated using the mediation R package (36) to calculate the average direct effect (ADE), the average causal mediation effect (ACME), the total effect (TE), and the proportion of mediated effect (PME). This effect is estimated by conducting 10,000 simulations using a quasi-Bayesian Monte Carlo method based on normal approximation (37).

## Results

The three main dietary patterns identified by factor analysis and the factor loadings for each dietary pattern are presented in Table 2. Factor 1 was characterized by vegetables, tubers, nuts, corn, legumes and its products, and was named “the vegetable-rich pattern.” The “animal-food pattern” (factor 2) was loaded heavily for poultry and its products, meat and its products, animal organ, beverages, fish and shellfish, wheat and its products and rice and its products. Factor 3 (the “prudent dietary pattern”) was marked by high intakes of fruits, whole grains products, dairy products, eggs, wheat and its products, and ethnic foods, with inverse loading for rice and its products. These three dietary patterns explained 24.6% of the total variation in dietary intake (11.1%, 6.9%, and 6.6% for factor 1, factor 2, and factor 3, respectively).

**Table 2:** Orthogonally rotated factor loadings for three dietary patterns derived from factor analysis^a^.

Food or food groups	Factor 1	Factor 2	Factor 3
	Vegetable food	Animal food	Prudent
Rice and its products	0.25	**0.32**	**−0.39**
Noodles	0.20	0.06	−0.04
Wheats and its products	0.14	**0.34**	**0.38**
Whole grains products	0.04	0.08	**0.47**
Ethnic foods	0.03	0.04	**0.35**
Corn	**0.41**	−0.14	0.04
Tubers	**0.55**	0.06	0.10
Sweet potato	**0.35**	−0.20	−0.16
Legumes and its products	**0.41**	0.25	0.03
Dark Vegetables	**0.65**	0.11	−0.01
Light-colored vegetables	**0.54**	0.20	0.09
Fungi and algae	**0.37**	0.29	0.29
Fruits	0.16	−0.02	**0.48**
Nuts	**0.42**	−0.22	0.00
Meat and its products	0.18	**0.56**	−0.28
Poultry and its products	0.08	**0.67**	0.11
Animal organ	−0.04	**0.51**	−0.03
Fish and shellfish	0.15	**0.46**	0.07
Eggs	0.28	−0.04	**0.38**
Preserved eggs	0.20	0.09	0.14
Pickled vegetables	0.19	0.18	−0.28
Dairy products	0.01	−0.06	**0.46**
Tea and coffee	0.14	0.01	0.06
Beverages	−0.15	**0.50**	0.12
% Variance explained	11.1%	6.9%	6.6%

a Factor loadings ≥ 0.30 or ≤ −0.30 are bolded.

General characteristics of study participants according to tertiles of each dietary pattern are presented in Table 3. The mean age of the study population was 45.0 ± 13.7 years. 46.5% of the study participants were female, with mean values of SBP and DBP being 123.7 ± 16.5 and 78.1 ± 10.6 mmHg, respectively. The vegetable-rich pattern was positively associated with age, SBP, and DBP. Participants with a higher score for vegetable-rich pattern were more likely to drink alcohol compared with those with a lower score. There were no associations between vegetable-rich pattern and gender, BMI, WC, BF% or smoking status. Animal-food pattern score was inversely associated with age and BF%, and positively associated with BMI and WC. Participants with a higher score for the animal food pattern were more likely to smoke or drink alcohol than participants with a lower score. Prudent dietary pattern was inversely associated with BMI, WC, SBP, DBP, and a greater percentage of not current smokers. All three patterns were positively correlated with energy intake.

**Table 3:** Participant characteristics according to categories of dietary patterns^a^.

	All subjects	Vegetable-rich pattern			Animal-food pattern			Prudent dietary pattern		
		T1	T3	*p*	T1	T3	*p*	T1	T3	*p*
*N*	6,849	2,283	2,283		2,283	2,283		2,283	2,283	
Age (y)	45.0 ± 13.7	41.6 ± 13.7	47.5 ± 13.3	<0.0001	52.0 ± 13.7	38.5 ± 12.1	<0.0001	46.2 ± 11.0	44.9 ± 15.8	0.09
**Gender (%)**										
Female	46.5	55.5	44.7	0.4	74.2	21.1	<0.0001	42.0	51.5	0.1
Male	53.5	44.5	55.3		25.9	78.9		58.1	48.5	
BMI (kg/m^2^)	24.0 ± 3.1	23.8 ± 3.2	24.2 ± 3.1	0.2	23.5 ± 3.0	24.3 ± 3.3	0.02	24.6 ± 3.1	23.1 ± 3.0	<0.0001
WC	85.6 ± 8.6	85.2 ± 8.9	85.8 ± 8.2	0.3	84.5 ± 8.3	86.5 ± 9.1	0.007	86.9 ± 8.2	83.6 ± 8.4	0.02
BF%	27.2 ± 6.6	26.7 ± 6.4	27.2 ± 6.7	0.8	30.3 ± 6.0	24.4 ± 5.7	<0.0001	27.3 ± 6.8	27.0 ± 6.4	0.1
**Alcohol consumption (%)**										
Yes	52.3	53.8	57.0	0.048	34.3	70.9	<0.0001	52.1	49.8	0.5
No	47.8	46.2	43.0		65.7	29.1		47.9	50.2	
*Smoking status (%)*										
Current smoker	23.8	25.4	21.9	0.7	8.9	38.4	<0.0001	32.2	15.6	0.0001
Non-current smoker	76.2	74.6	78.1		91.1	61.6		67.8	83.4	
Energy intake (kcal/d)	1376.0 ± 523.5	1100.3 ± 475.0	1698.8 ± 532.1	<0.0001	1154.7 ± 503.0	1687.8 ± 526.0	<0.0001	1,304 ± 489.3	1558.5 ± 586.2	<0.0001
Physical activity (MET-h/wk)	17.5 ± 9.8	14.7 ± 7.0	18.9 ± 8.4	0.08	18.9 ± 9.9	16.8 ± 7.7	<0.0001	16.8 ± 7.0	17.5 ± 9.6	<0.0001
SBP (mmHg)	123.7 ± 16.5	120.6 ± 15.6	126.0 + 16.8	0.0001	124.0 + 18.0	123.7 + 14.9	0.7	127.4 ± 17.7	120.9 ± 16.2	0.0007
DBP (mmHg)	78.1 ± 10.6	77.2 ± 10.1	79.0 ± 10.9	0.04	76.7 ± 10.2	79.2 ± 10.5	0.07	81.2 ± 10.3	76.2 ± 10.8	0.0006

a Data were presented as means ± SD or percent; T1, tertile 1, including individuals with lowest dietary pattern score; T3, tertile 3, including individuals with highest dietary pattern score; difference in proportions were measured by Chi-square analysis (categorical variables) or linear trends were measured by analysis of variance (continuous variables) across tertiles.

SBP, systolic blood pressure; DBP, diastolic blood pressure; BMI, body mass index; WC, waist circumstance; BF%, body fat percentage.

Nutrient intakes across tertile of each dietary pattern are shown in Table 4. A higher vegetable-rich pattern score was associated with a higher protein, potassium, calcium, fiber, and most vitamins, including Vitamin A, Vitamin B1, Vitamin B2, Vitamin B3, Vitamin C, and Vitamin E. A higher animal food pattern score was associated with a higher intake of protein and vitamin A. The carbohydrate, calcium, Vitamin B1, Vitamin C, Vitamin E, and Fiber intake were significantly lower as the animal food pattern scores increased. Prudent pattern score was positively correlated with carbohydrate, potassium, calcium, Vitamin B1, Vitamin B2, Vitamin C, Vitamin E, and fiber, and inversely correlated with fat, protein, and vitamin B3.

**Table 4:** Mean nutrient intakes for tertile of dietary pattern score^a^.

	Vegetable-rich pattern			Animal-food pattern			Prudent dietary pattern		
	T1	T3	*p*	T1	T3	*p*	T1	T3	*p*
Carbohydrate (%E)	57.0 ± 1.2	56.2 ± 1.2	0.2	58.2 ± 1.2	54.2 ± 1.2	<0.0001	55.5 ± 1.1	56.8 ± 1.1	0.002
Fat (%E)	26.1 ± 1.1	25.5 ± 1.0	0.8	25.6 ± 1.0	26.7 ± 1.0	0.3	26.4 ± 0.8	26.3 ± 0.8	0.04
Protein (%E)	17.0 ± 0.2	18.3 ± 0.4	<0.0001	16.2 ± 0.4	19.2 ± 0.4	<0.0001	18.1 ± 0.4	17.0 ± 0.4	<0.0001
K (mg/1000 kcal)	1119.5 ± 38.8	1527.1 ± 38.9	<0.0001	1343.5 ± 43.8	1286.7 ± 45.2	0.2	1190.9 ± 39.1	1380.2 ± 39.5	<0.0001
Ca (mg/1000 kcal)	267.8 ± 15.7	314.6 ± 15.7	<0.0001	324.8 ± 15.7	259.6 ± 16.1	0.0002	223.2 ± 13.4	338.1 ± 13.5	<0.0001
Mg (mg/1000 kcal)	163.3 ± 4.9	216.7 ± 5.4	<0.0001	199.4 ± 5.0	177.5 ± 4.1	<0.0001	179.6 ± 5.4	190.5 ± 5.5	0.006
Vitamin A (mg RAE/1000 kcal)	313.3 ± 29.5	424.0 ± 29.6	<0.0001	344.5 ± 30.1	405.4 ± 30.9	<0.0001	339.6 ± 27.8	359.9 ± 28.0	0.2
Vitamin B_1_ (mg/1000 kcal)	0.47 ± 0.01	0.55 ± 0.01	<0.0001	0.54 ± 0.01	0.47 ± 0.01	<0.0001	0.47 ± 0.01	0.53 ± 0.01	<0.0001
Vitamin B_2_ (mg/1000 kcal)	0.62 ± 0.02	0.69 ± 0.02	<0.0001	0.67 ± 0.02	0.65 ± 0.02	0.07	0.58 ± 0.02	0.71 ± 0.02	<0.0001
Vitamin B_3_ (mg/1000 kcal)	11.3 ± 0.3	12.3 ± 0.3	0.001	9.8 ± 0.3	13.8 ± 0.3	<0.0001	13.6 ± 0.3	10.1 ± 0.3	<0.0001
Vitamin C (mg/1000 kcal)	59.8 ± 5.9	103.0 ± 5.9	<0.0001	87.9 ± 6.3	74.2 ± 6.4	0.001	66.2 ± 5.6	92.6 ± 5.7	<0.0001
Vitamin E (mg/1000 kcal)	7.7 ± 0.5	9.8 ± 0.5	<0.0001	10.5 ± 0.5	7.2 ± 0.5	<0.0001	7.4 ± 0.4	9.8 ± 0.5	<0.0001
Fiber (g/1000 kcal)	7.4 ± 0.4	10.7 ± 0.4	<0.0001	9.9 ± 0.4	8.0 ± 0.4	<0.0001	7.6 ± 0.4	10.0 ± 0.4	<0.0001

a Values are least-squares means±SD for tertile 1 and tertile 3 of dietary pattern score, from models adjusting for age, gender, energy intake; *p*-values refer to general linear regression.

E, energy; K, potassium; Ca, calcium; Mg, magnesium.

Table 5 presents the associations of tertiles of each dietary pattern with SBP and DBP. Multiple linear regression analysis showed that prudent dietary pattern was inversely related to SBP (*β* = −1,73, *p* for trend =0.004) and DBP (*β* = −1.20, *p* for trend =0.002) after adjustment for age and gender (model 1). Further adjustment for smoke state, alcohol consumption, energy intake and physical activity (model 2) did not change these inverse associations (for SBP, *β* = −2.30, *p* for trend =0.0003; for DBP, *β* = −1.44, *p* for trend =0.0006). In models 3–5, the strength of relation between prudent dietary pattern score with SBP (*β* = −1.35, *p* for trend =0.03, model 3; *β* = −1.65, *p* for trend =0.007, model 4; *β* = −1.89, *p* for trend =0.02, model 5) and DBP (*β* = −0,79, *p* for trend =0.045, model3; *β* = −0.99, *p* for trend =0.01, model 4; *β* = −1.14, *p* for trend =0.004, model 5) was attenuated but remained significant with additional adjustment for body composition. While the vegetable-rich pattern and animal-food pattern were not associated with either SBP and DBP in all models (all *p* > 0.05).

**Table 5:** Association between dietary patterns and blood pressure^a^.

	SBP		DBP	
	*β* (SE)	*p _for trend_*	*β* (SE)	*p*_for trend_
*Vegetable-rich pattern*				
Model 1^b^	1.06 (0.62)	0.09	0.38 (0.40)	0.3
Model 2^c^	1.12 (0.76)	0.1	0.56 (0.50)	0.3
Model 3^d^	0.98 (0.71)	0.2	0.42 (0.46)	0.4
Model 4^e^	1.29 (0.72)	0.07	0.63 (0.47)	0.2
Model 5^f^	1.30 (0.73)	0.08	0.64 (0.47)	0.2
*Animal-food pattern*				
Model 1^b^	0.78 (0.67)	0.2	0.50 (0.44)	0.3
Model 2^c^	0.92 (0.76)	0.2	0.73 (0.50)	0.1
Model 3^d^	0.04 (0.72)	0.9	0.12 (0.47)	0.8
Model 4^e^	−0.23 (0.73)	0.8	−0.06 (0.48)	0.9
Model 5^f^	−0.05 (0.74)	0.9	0.00 (0.48)	0.9
*Prudent dietary pattern*				
Model 1^b^	−1.73 (0.59)	**0.004**	−1.20 (0.39)	**0.002**
Model 2^c^	−2.30 (0.64)	**0.0003**	−1.44 (0.42)	**0.0006**
Model 3^d^	−1.35 (0.61)	**0.03**	−0.79 (0.39)	**0.045**
Model 4^e^	−1.65 (0.61)	**0.007**	−0.99 (0.39)	**0.01**
Model 5^f^	−1.89 (0.62)	**0.002**	−1.14 (0.40)	**0.004**

a Values are estimate (SE, standard error). Linear trends (*p_for trend_*) were obtained with SBP, DBP as continuous variables. Significant data are in bold.

b Model 1 adjusted for age, gender.

c Model 2 adjusted for variables in model 1 and alcohol consumption, current smoker, energy intake, physical activity.

d Model 3 adjusted for variables in model 2 and BMI.

e Model 4 adjusted for variables in model 2 and WC.

f Model 5 adjusted for variables in model 2 and BF%.

SBP, systolic blood pressure; DBP, diastolic blood pressure; BMI, body mass index; WC, waist circumstance; BF%, body fat percentage.

**Figure 2:**
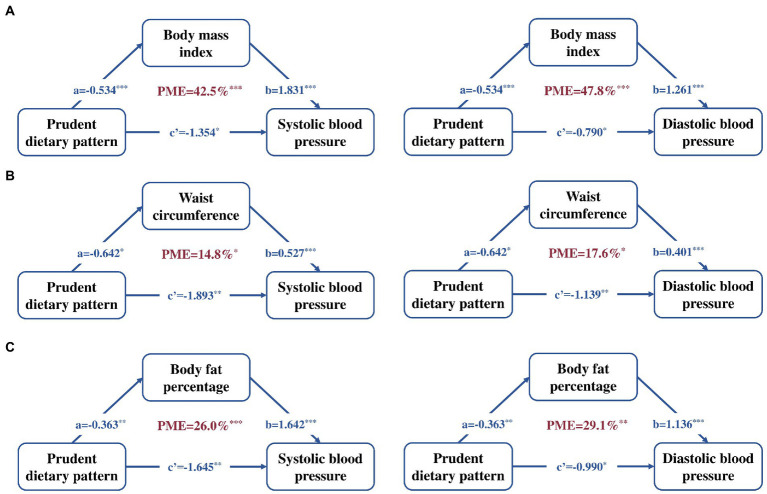
Mediation linkages among the prudent patterns scores (continuous exposure), blood pressure (continuous outcome) and body composition (continuous mediator). Adjusted for age, gender, alcohol consumption, current smoker, energy intake and physical activity. **(A)** BMI as the independent variable, **(B)** WC as the independent variable, and **(C)** BF% as the independent variable. Regression coefficients are presented, with the path a representing the effect of prudent patterns scores on body composition, path b representing the effect of body composition on blood pressure, path c’ representing the direct effect of prudent patterns scores on blood pressure, The PME reflects the proportion of the total effect of the prudent patterns scores on the blood pressure that is explained by body composition. *p < 0.05, **p < 0.01, ***p < 0.001. BMI, body mass index; DBP, diastolic blood pressure; PME, the proportion of mediated effect; SBP, systolic blood pressure; WC, waist circumstance; BF%, body fat percentage.

Since the strength of the association between prudent dietary pattern scores with SBP and DBP was attenuated by additional adjustment for body composition., we conducted a mediation analysis between prudent dietary pattern, body composition., and blood pressure (Figure 2). The results of mediation analyses indicated that BMI WC and BF% partially mediated the association between prudent dietary pattern and blood pressure, respectively. 42.5%/47.8% of the association between prudent dietary pattern scores and SBP/DBP was mediated by BMI (Figure 2A). In Figure 2B, the WC contributed to 14.8%/17.6% of the total effect of prudent dietary pattern on SBP/DBP. The between prudent dietary pattern scores and SBP/DBP was explained 26.0%/29.1% by BF% (Figure 2C).

## Discussion

In this sample of Chinese adults, we identified the prudent dietary pattern, vegetable-rich pattern, and animal-food pattern, among them, only the prudent pattern was found to be inversely associated with SBP and DBP. In addition, BMI, WC and BF% contributed to, respectively, 42.5%/47.8, 14.8%/17.6 and 26.0%/29.1% of these associations of prudent pattern with SBP/DBP.

Compared with studies focusing on single nutrients or foods, our findings about the negative association between the prudent pattern with blood pressure have a more direct public health implication. Each unit increase in prudent pattern scores was associated with a 2.30 mmHg lower SBP and 1.44 mmHg lower DBP after adjusted for age, gender, alcohol consumption, current smoker, energy intake and physical activity. Although this change is relatively small, it is significant on a population level, for example, a 5 mm Hg reduction in blood pressure would prevent around 200,000 deaths per year among individuals younger than 70 years of age (38) and associated with around 4% lower mortality from coronary heart disease (39). Our result was in line with the study conducted in Korean adults aged 20–64 years (40). Moreover, we tried to find some clues in the loading foods of prudent pattern, and fruits were independently associated with blood pressure (data not shown) in our sample. Additionally, high-correlated nutrients, calcium (41), potassium (42), and magnesium (43) in prudent pattern were found to play crucial roles in the prevention of hypertension. Therefore, the impact of prudent pattern on lowing DBP and SBP could be considered according to a synergistic effect of these foods and nutrients. The prudent dietary pattern characterized by higher intakes of fruits, whole grains and low intakes of red meat was similar to the DASH diet, which is a healthy eating pattern have demonstrated positive effects on blood pressure (44).

Body composition played a critical mediating role in the association between prudent dietary pattern and blood pressure. Interestingly, the mediating effect of BMI is the largest with approximately half of the total effect of prudent pattern on blood pressure (42.5% for SBP and 47.8% for DBP), which might be partly explained by the evidence that measures of general adiposity had been found to be more strongly related to blood pressure than measures of central adiposity in Chinese adults (19, 45). These findings showed an independent effect of prudent pattern on reducing blood pressure, which could not be ignored in the first place, and provided evidence for diet recommendations to prevent elevated blood pressure. In addition, the disclosed mediator effect of body composition indicated that obesity status seemed to be important for the dietary recommendation to hypertension control and reinforced the need to maintain a healthy weight, WC, and body fat in public health practice. However, the underlying biologic mechanisms remains unknown, and further research are needed to address the prospective interplay among dietary patterns, body composition, and blood pressure in Chinese adults.

Although some studies have suggested that a vegan or vegetarian diet may be protective against obesity, type 2 diabetes, or CVD (46, 47), vegetable-rich pattern in our study did not significantly affect blood pressure. This discrepancy might lie in the characteristic of our sample. Participants in the highest tertile had an approximately 14% higher age compared to those in the lowest tertile, and most of the elderly tend to change their eating habits. Moreover, it cannot be denied that the efficacy of BP lowering using the vegetable-rich dietary modification was inconsistent recently (44). For animal food pattern, our finding is consistent with the results reported in the previous study (48) among Chinese population, but its positive association with blood pressure in subjects from other countries (49, 50), suggesting that the effect of the animal food pattern on blood pressure may be race specific. Further prospective research is needed to confirm the impact of these dietary patterns on blood pressure.

The strengths of our study included its representative study sample and detailed measurement of blood pressure, dietary and anthropometric measurements with the ability to adjust for several major potential confounders. Meanwhile, we considered the mediation effect of body composition to optimize public health practice. Nevertheless, our study had several limitations. Given the cross-sectional design of this study, we were unable to evaluate the causal relationship between dietary patterns and blood pressure. Future prospective cohort studies are warranted to verify our findings. In addition, there was a lack of information on foods (e.g., types of meats, beverages, cooking methods, and seasoning), which play an important role in the regulation of blood pressure (51–53), so it is difficult to characterize dietary patterns in more specific details. Furthermore, although we have adjusted for demographics and lifestyle factors, residual confounding unmeasured factors might be present.

In this study, prudent dietary pattern characterized by higher intake of fruits, whole grains products, dairy products, eggs, and wheat and its products, and lower intakes of rice and its products was associated with lower SBP and DBP among Chinese adults. This association was partially explained by body composition.

## Data availability statement

The raw data supporting the conclusions of this article will be made available by the authors, without undue reservation.

## Author contributions

GC conceived the project. MC and YX performed the analyses and wrote the manuscript. MC performed the initial data analyses. XW and SS coordinated the study centers. GC supervised the study. All authors contributed to the article and approved the submitted version.

## Funding

The study was funded by Study of Diet and Nutrition Assessment and Intervention Technology (no. 2020YFC2006300) from Active Health and Aging Technologic Solutions Major Project of National Key R&D Program, and Key R&D Project in Sichuan Province (no. 23ZDYF2599).

## Conflict of interest

The authors declare that the research was conducted in the absence of any commercial or financial relationships that could be construed as a potential conflict of interest.

## Publisher’s note

All claims expressed in this article are solely those of the authors and do not necessarily represent those of their affiliated organizations, or those of the publisher, the editors and the reviewers. Any product that may be evaluated in this article, or claim that may be made by its manufacturer, is not guaranteed or endorsed by the publisher.
